# Hidden Armour: The Passive Protective Function of Caudal Osteoderms in Snakes

**DOI:** 10.1002/jmor.70034

**Published:** 2025-02-22

**Authors:** Petra Frýdlová, Jan Dudák, Veronika Tymlová, Jan Žemlička, Jiří Moravec, Daniel Frynta

**Affiliations:** ^1^ Department of Zoology, Faculty of Science Charles University Prague Czech Republic; ^2^ Institute of Experimental and Applied Physics Czech Technical University in Prague Prague Czech Republic; ^3^ Department of Zoology National Museum Prague Czech Republic

**Keywords:** µCT, antipredatory strategy, dermal armour, osteoderms, Squamata

## Abstract

Dermal armour, consisting of bony dermal structures known as osteoderms (ODs), is widespread in squamate reptiles. However, in some limbless taxa such as snakes, ODs are rare, probably due to a trade‐off between mechanical protection and the demands of locomotion and consumption of large prey. Recent findings of ODs restricted to the distal body regions of sand boas (*Eryx*, Erycidae) challenge this paradigm, suggesting they provide passive mechanical protection against aggressive prey without significantly impairing locomotion. Building on these findings, we have continued the search and identified three additional snake species that have well‐developed caudal ODs, including the first‐ever discovery of ODs in shield‐tailed snakes (Uropeltidae). In these fossorial species, which are characterised by their unique tail morphology, ecological adaptations and colouration, the ODs at the tail tip may serve as passive protection against predators. However, an alternative role in locomotion or occasional phragmosis cannot be ruled out. In the Javelin sand boa (*Eryx jaculus*), the ODs are hypothesised to function as a mechanical defence against aggressive prey. These results highlight the functional and evolutionary plasticity of ODs and emphasise the urgent need for further studies on their specific role and adaptive significance in the ecology and evolution of snakes.

## Graphical abstract

Well‐developed caudal osteoderms were discovered in shield‐tailed snakes (Uropeltidae). Dermal armour at the tail tip likely provides passive protection against predators, though roles in locomotion or phragmosis cannot be excluded. Our findings emphasise the functional and evolutionary versatility of osteoderms and highlight the need for further research into their adaptive significance in snake ecology and evolution.

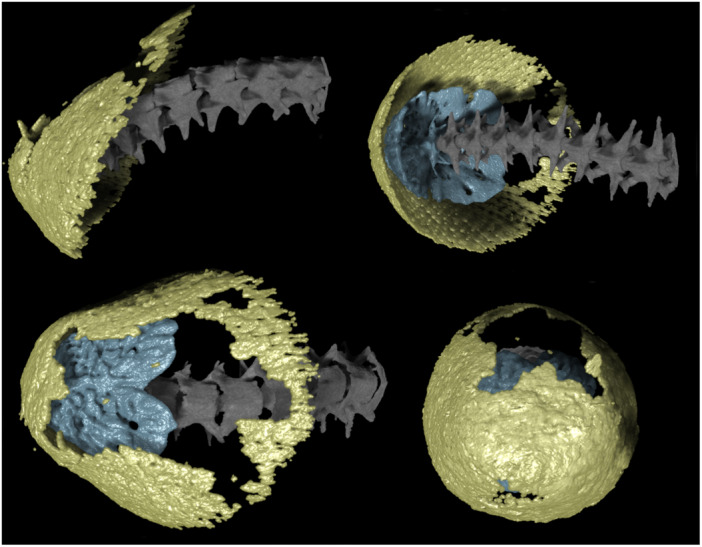

## 1 Introduction

Dermal bony plates, or osteoderms (ODs), are calcified organs formed during the ontogeny directly within the skin (Gadow 1901; Francillon‐Vieillot et al. 1990). They were described in the majority of extinct (stem‐amniotes, nonavian dinosaurs, archosaurs) as well as extant (amphibians, turtles, crocodylians, squamates and mammals) tetrapod lineages (for a review see Vickaryous and Sire 2009 and references herein). Nevertheless, the highest prevalence is undoubtedly in squamate lizards where the greatest diversity in its morphology and distribution was demonstrated and reviewed recently (Williams et al. 2022). From the distribution of ODs across Squamata's phylogenetic tree, there are two clades (Scincomorpha and Anguimorpha) with a high frequency of dermal armour (Williams et al. 2022). On the other hand, the presence of ODs is uncommon in Lacertoidea and very rare in Gekkota and Iguania (Williams et al. 2022). Moreover, dermal bony plates were thought to be absent in snakes and amphisbaenians until recently (Williams et al. 2022).

However, in 2023, Frýdlová et al. (2023) examined 27 snake species covering the most basal and advanced lineages and described the presence of dermal armour in four species of sand boas (*Eryx*, Erycidae). ODs in sand boas, revealed by the µCT technique, are tiny bony elements of vermiform‐like shape on the dorsal and lateral parts of the body and rod‐like shape on the ventral part. They are organised in a regular pattern resembling the pattern of scales. Their distribution is on the caudal part of the body near the cloaca opening and on the whole tail but only in adult individuals. Even though the functional morphology was not tested in this study, it was hypothesised to serve as a defensive armour against aggressive prey.

Mechanical defence preventing physical damage is the most common and easiest explanation of the dermal armour in current (Schucht et al. 2020; Vickaryous et al. 2015; Yang et al. 2013; Chintapalli et al. 2014) as well as the older literature (Moss 1972), even though also other theories as, for example, calcium storage (Paluh et al. 2017; Vidal et al. 2017), locomotion (Buchwitz and Voigt 2010), thermoregulation (Seidel 1979; Farlow et al. 2010; Broeckhoven et al. 2017b) and biomechanics (Marghoub et al. 2023) were proposed. Phylogenetically informed comparative studies considering covariation between OD expression, distribution or morphology and ecological factors such as climate put forward the idea that ODs most probably have a multifunctional nature (Clarac et al. 2019; Broeckhoven and du Plessis 2022). Currently, many studies have appeared focusing on the functional morphology of ODs testing this basic protective hypothesis (Iacoviello et al. 2020; Kéver et al. 2022; Marghoub et al. 2023).

A prime example of mechanical body defence is usually in an antipredatory context (McDonough and Loughry 1997; Bergstrom and Reimchen 2003). Paradoxically, the poor development of ODs in reptile juveniles, which are much more susceptible to predation (English 2018) than adults, and their subsequent development in adulthood, is puzzling. It would be much more beneficial to develop the armour earlier in ontogeny and especially for reptiles, given that they usually do not have developed parental care (Shine 1988). The reason may be that the costs of bearing armour are high. A correlated reduction in encephalisation has been proven in armoured armadillos (Stankowich and Romero 2017), whereas girdled lizards (Losos et al. 2002) or sticklebacks (Andraso 1997; Bergstrom 2002) suffer from a negative effect of armour on locomotor performance. It seems that reptile juveniles rather than relying on armour, prefer to use another defensive mechanism like cryptic colouration (Ortega et al. 2014) and adjustment of their antipredatory behaviour (Greene 1988; Landová et al. 2013).

Another context of mechanical defence was reviewed recently by Broeckhoven (2022). It is the fighting advantage hypothesis suggesting that ODs are developed to protect the body against conspecifics. This can be supported by the fact that it is developed in sexually mature animals and its expression can be sexually dimorphic (Reimchen et al. 2016; Broeckhoven et al. 2018). Moreover, the distribution of ODs is often more expressed in the body regions that are targeted by conspecifics during the combats (Broeckhoven et al. 2017a).

Mechanical defence can work against aggressive prey as hypothesised in geckos (Hoofien 1962) and sand boas (Frýdlová et al. 2023). The skin of the white‐spotted wall gecko (*Tarentola annularis*) is reinforced with an OD cover and thus possibly impenetrable to the sting of scorpions. Sand boas are equipped with a variety of morphological modifications and antipredatory strategies to protect their body (thoroughly summarised in Frýdlová et al. 2023). The skin surface on the tail of some sand boas is protected with ODs (Frýdlová et al. 2023). Their tail resembles the head (so‐called ‘two‐headed’ snakes, O'Shea 2018) and postcaudal vertebrae are highly modified (Sood 1941; Bogert 1968; Szyndlar and Schleich 1994). Sand boas prey on rodent pups in underground burrows. They are usually able to defend themselves effectively through active biting and constriction. However, they become vulnerable during the process of swallowing. It is assumed that both internal (increased volume of highly modified caudal vertebrae) and external (ODs distributed on the tail) mechanical reinforcements of the body provide them with passive defence against the combative parents of their prey.

Apart from direct protection from penetration, the mechanical defence can work also indirectly. ODs can reinforce spikes and keels, which discourage predators from attacks, or they can complicate prey removal from a burrow (e.g., Chapple 2003; Maliuk et al. 2024; Stanley et al. 2016). A special case is a so‐called phragmosis when the removal from the burrow is highly complicated as it is closed by some modified part of the body (Wheeler 1927). This was described in cordylid (Cooper et al. 2000) and scincid lizards (Hutchinson et al. 1994), frogs (Jared et al. 2005) and armadillos (Krmpotic et al. 2024; Schmidt 1955). According to the ecology and morphological adaptations, this seems to be a feasible scenario for the shield‐tailed snakes.

Our previous study (Frýdlová et al. 2023) examined only a subset of the remarkable biodiversity within the snake lineage, which currently comprises over 4154 species according to The Reptile Database (Uetz et al. 2024). Our findings revealed the presence of ODs exclusively in the family Erycidae. Notably, ODs were absent in the Javelin sand boa (*Eryx jaculus*), despite their presence in closely related species (*E. tataricus, E. miliaris, E. colubrinus* and *E. conicus* Frýdlová et al. 2023). To investigate this inconsistency further, we collected two additional, larger specimens of *E. jaculus* for examination. We also focused on the Rubber boa (*Charina bottae*), a species exhibiting postcaudal vertebral modifications similar to those observed in sand boas (Bogert 1968). A mechanical defence function against combative prey was hypothesised for this species (Hoyer 1974; Nussbaum and Hoyer 1974; Hoyer and Stewart 2000b). Closely related sand and rubber boas share several other characteristics, including semi‐fossorial or fossorial lifestyle (Esquerré and Scott Keogh 2016), a diet consisting of rodent pups, ‘two‐headed’ morphology and tail display behaviours that divert attention to the distal part of the body (Greene 1973). These shared characteristics strongly suggest that the tails of both groups are under significant evolutionary pressure.

To expand our comparative framework, we included two additional closely related groups of snakes in our study: shield‐tailed snakes (family Uropeltidae) and Asian Pipe snakes (family Cylindrophiidae), both belonging to the Uropeltoidea superfamily. Uropeltoids are small, secretive burrowing henophidian snakes closely related to the Booidae superfamily (Graham Reynolds et al. 2014). Representing one of the least‐studied snake groups in South Asia (Pyron et al. 2016), they share several ecological and behavioural traits with sand boas, such as fossorial habits (Mattison 2015), ‘two‐headed’ morphology and tail displays (Gans 1986). Despite these similarities, uropeltoids exhibit unique biological characteristics in comparison to sand boas. Shield‐tailed snakes specialise in earthworms (Smith 1943), and Asian Pipe snakes can prey on elongated vertebrates, including other snakes, caecilians and eels (Himstedt et al. 2003; Priyadarshana et al. 2016). Their locomotion is highly specialised (Gans et al. 1978), and their cryptic dorsal colouration contrasts sharply with conspicuous ventral patterns (Cyriac and Kodandaramaiah 2019). We hypothesise that the selection pressure driving the potential development of ODs in uropeltoids may differ from those affecting sand boas.

To conduct our research, we employed µCT scanning as a nondestructive method for detecting and visualising hard tissue. Collaborating with the National Museum of the Czech Republic allowed us access to rare and less commonly studied species that are not typically maintained by private breeders or zoological gardens.

## 2 Materials and Methods

### 2.1 Specimens

We examined three specimens of two species of shield‐tailed snakes from the family Uropeltidae *Rhinophis homolepis* (Hemprich, 1820) and *Uropeltis macrolepis* (Peters, 1862), one Black pipe snake *Cylindrophis melanotus* Wagler, 1828 from the family Cylindrophiidae, one Northern Rubber boa *Charina bottae* (Blainville, 1835) from the family Charinaidae and two Javelin sand boas *Eryx jaculus* (Linnaeus, 1758) from the family Erycidae. All details concerning the material are summarised in Table 1. Samples were preserved in an alcohol or formaldehyde solution. We measured the total body length of all specimens to the nearest 1 mm and expressed it as an absolute (TOL) and a relative (TOL_rel_) value. The latter represents a percent ratio of the TOL of the examined specimen to the maximum TOL reported in the literature for the particular species (and sex if available); that is, TOL_rel_ 100% means the maximum reported size. As for shield‐tailed snakes are only very limited data about their body size in literature, TOL_rel_ should be taken as indicative only. The values for absolute and relative TOLs and references are summarised in Table 1. We completed our results with data about the ecology (fossorial/semi‐fossorial) and behaviour (tail displays present/absent).

**Table 1:** The list of studied specimens with details about the presence of ODs, caudal vertebrae modifications and other life‐history variables.

Species	Locality	ODs	CVM	Tail displays	TOL (mm)	TOL_rel_ (%)	Sex	Fossoriality
Sample number	Year							
*Rhinophis homolepis*	Ceylon	1	1	0	183	65.4^(4)^	—	F^(8)^
(NMP‐P6V 73420)	1885							
*R. homolepis*	Ceylon	1	1	0	202	72.1^(4)^	—	F^(8)^
(NMP‐P6V 73419)	1885							
*Uropeltis macrolepis*	India	1	0	0	196	65.3^(4)^	Male	F^(8)^
(NMP‐P6V 73421)	1935							
*Charina bottae*	California	0	1	1^(1)^	486	89.0^(5)^	Female	SF^(8)^
(NMP‐P6V 32794)								
*Eryx jaculus*	Captive bred	1	1	1^(2)^	622	88.1^(6)^	Female	F^(8)^
(CUNI‐817)								
*E. jaculus*	Captive bred	1	1	1^(2)^	600	85.0^(6)^	Female	F^(8)^
(CUNI‐818)								
*Cylindrophis melanotus*	Celebes, Rantepao	0	0	1^(3)^	430	73.4^(7)^	—	SF^(9)^
(CUNI‐401)								

Abbreviations: 0, absent; 1, present; CVM, caudal vertebrae modifications; F, fossorial; OD, osteoderm; SF, semi‐fossorial; TOL, total body length; TOLrel, relative total body length.

*Source:* (1) Keegan (1943); (2) Stemmler (1958); (3) Deraniyagala (1955); (4) Smith (1943); (5) Hoyer and Stewart (2000a); (6) Eskandarzadeh et al. (2018); (7) Amarashinghe et al. (2015); (8) Esquerré and Scott Keogh (2016); (9) Riendriasari et al. (2008).

### 2.2 Imaging Techniques

The samples were scanned at the micro‐CT laboratory of the Institute of Experimental and Applied Physics, Czech Technical University in Prague. The used system utilises a cone‐beam imaging geometry and for purposes of the presented work, it was equipped with micro‐focus X‐ray tube Hamamatsu L12531 and detector unit Dexela 1512. The source is characterised by a tungsten anode, accelerating voltage up to 110 kV and output power up to 16 W. The detector is a flat‐panel‐type device with a CsI micro‐columnar scintillation sensor pixelated into an array of 1944 by 1536 pixels with a pitch of 74.8 µm. Further, the system offers a motorised multiaxial positioning system that allows for setting a demanded imaging geometry and sample positioning during the scan.

The imaging geometry and the resulting voxel size were set individually for each sample based on its size and shape to utilise reasonably the detector field of view. The summary of the scan parameters is presented in ESM S1 (Table S1). The majority of the samples were scanned in a standard cone‐beam micro‐CT. Samples 817 and 818 were, however, scanned in a spiral imaging geometry, as the samples were too long for a standard scan. The CT reconstruction was conducted using a dedicated module of VG Studio MAX. The following analysis and visualisations were carried out using VG Studio MAX and ORS Dragonfly software, Version 2022.2 (Dragonfly 2022).

## 3 Results

**Figure 1:**
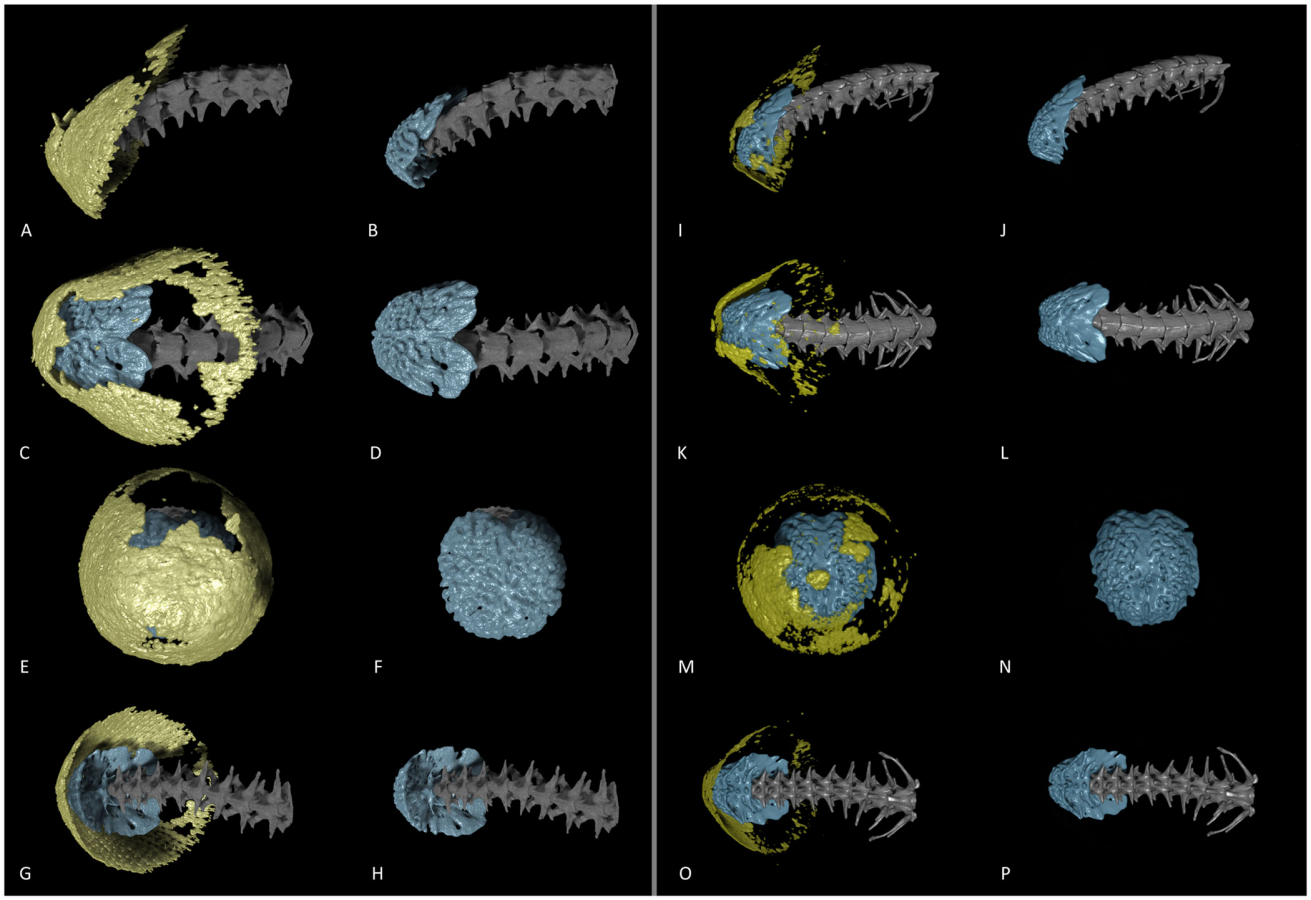
Visualisation of tail morphology of two individuals of Trevelyan's earth snake (*Rhinophis homolepis*). Conspicuous modification of the last vertebra (blue tip of the tail) is visible; the rest of the tail vertebrae are grey. Osteoderms (yellow cap) forming dermal armour are on the surface of the tail tip below the keratinised shield (not visible here). Dermal armour is a thin nearly continuous layer of the calcified tissue copying the surface of the modified caudal vertebra. Lateral view (A, B, I, J), dorsal view (C, D, K, L), caudal view (E, F, M, N) and ventral view (G, H, O, P). Compare the left side (A, C, E, G, I, K, M, O) with the dermal armour visible with the right side (B, D, F, H, J, L, N, P), where it was forced to be invisible to allow the detail view of the modified last caudal vertebra. NMP‐P6V 73419 specimen left, NMP‐P6V 73420 specimen right.

**Figure 2:**
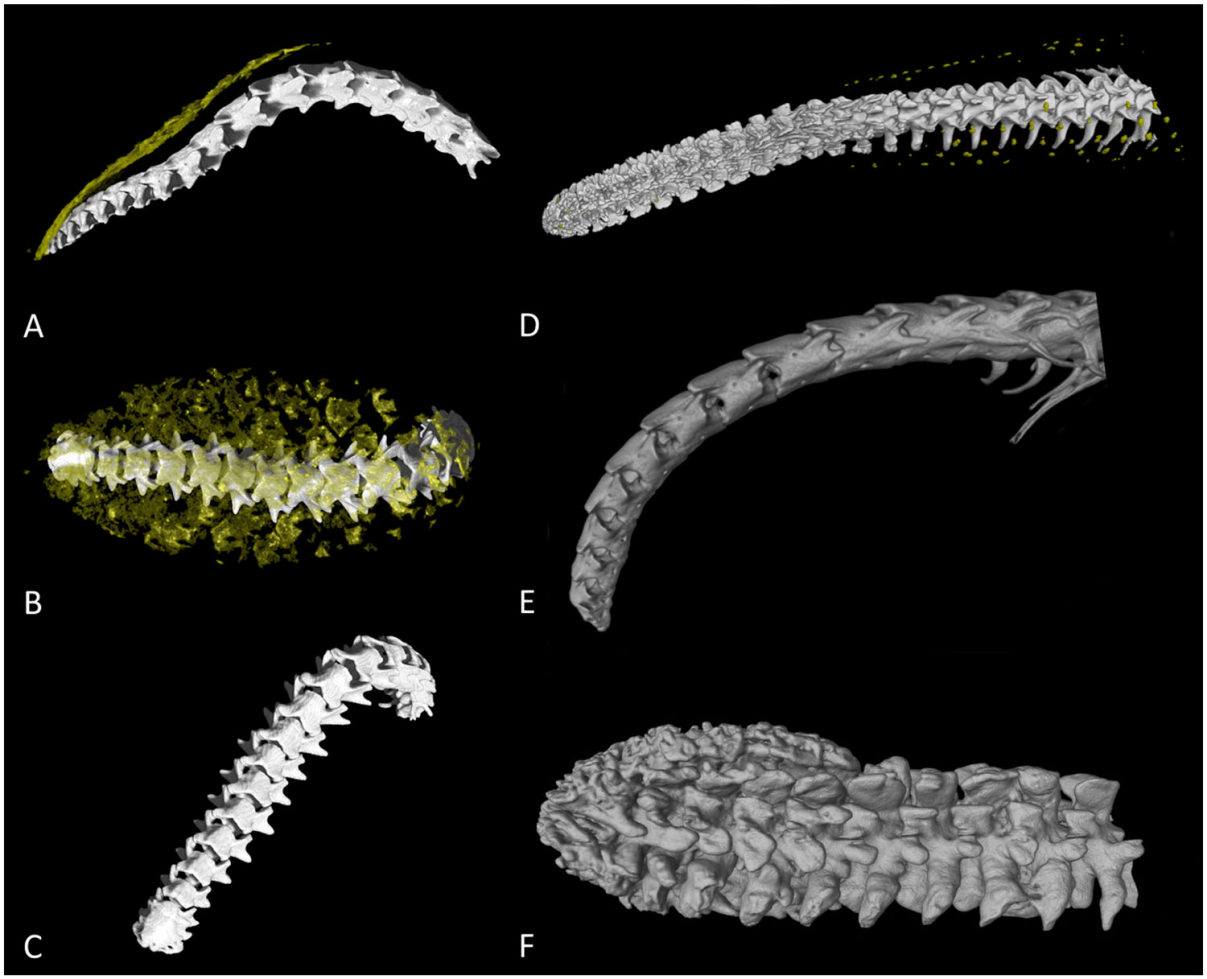
Visualisation of the tail morphology of several snake species. (A–C) The tail vertebrae of the Bombay earth snake (*Uropeltis macrolepis*) are gradually diminishing toward the tail tip with no visible modifications except the fusion of the last vertebrae. Osteoderms (yellow) are on the dorsal surface of the tail tip formed as a thin nearly continuous layer copying the surface below the keratinised shield. Lateral (A) and dorsal (B) view on the tail with visible osteoderms. The tail tip from the caudal side (C) with fused last vertebrae. (D) Tail morphology of Javelin sand boa (*Eryx jaculus*). The small yellow structures individually distributed across the surface of the tail inside the skin are osteoderms. Caudal vertebrae are highly modified. (E) Tail morphology of the Black pipe snake (*Cylindrophis melanotus*) and (F) Rubber boa (*Charina bottae*). We did not find the presence of ODs. The caudal vertebrae of the Black pipe snake are gradually diminishing toward the tip (E). In the Rubber boa, they are highly modified and almost fill the volume of the entire tail (F).

We examined rare species of snakes with similar ecological and/or behavioural characteristics to sand boas (tail resembling the head, presence of tail displays, caudal vertebrae modifications) to search for ODs. As the samples were mostly unique and very old specimens from the collections of the National Museum of the Czech Republic (NMP‐P6V), we employed only µCT as a nondestructive method for visualising mineralised tissue. We found ODs localised on the tail in three new species of snakes from two families. The most striking ODs as well as caudal vertebrae modifications were uncovered in Trevelyan's earth snake (*R. homolepis*). The inner morphology of basal caudal and cloacal vertebrae does not differ considerably from other snakes. The neural arch is depressed, haemapophyses are unpaired and appear in all caudal and cloacal vertebrae, lymphapophyses on four cloacal vertebrae are long and rounded, pleurapophyses are short but robust, and laterally directed throughout the caudal series consisting of eight to nine caudal vertebrae. Nevertheless, the posteriormost three to four caudal vertebrae are fused, enlarged and modified into a robust, symmetrical, highly mineralised structure (Figure 1, ESM S2 and S3) resembling the tail club knobs of prehistoric creatures. The dermal armour is on the surface of the tail tip below the shield. It is a thin continuous layer of the calcified tissue copying the surface of the tail club. The extent of dermal armour was similar in both specimens, which were according to the body size subadults (see Table 1 for TOL_rel_, and information about the body size of shield‐tailed snakes in Discussion). The shape of inner bony elements and the dense bulbous morphology of the tail tip resemble the tail club of ankylosaurs (Arbour and Currie 2015), glyptodons (Zurita et al. 2013) or turtles from the Meiolaniidae family (Sterli 2015), which used it most probably as a weapon for active defensive behaviour (Arbour and Zanno 2020). In contrast, Trevelyan's earth snakes use it rather for passive protection of the vulnerable tail tip. Other ODs were found on the tail of a subadult specimen of the Bombay earth snake (*U. macrolepis*). Caudal vertebrae of this species are rather typical as in other snake species (diminishing toward the tip). The neural arch is extremely depressed, haemapophyses are tiny and blunt in all caudal and cloacal vertebrae, and laterally directed pleurapophyses are very short and blunt throughout the caudal series. The posteriormost (approximately) three to four caudal vertebrae are fused (Figure 2C). We did not observe any other specific caudal modifications. Dermal armour was on the dorsal side of the tail directly below the tail shield (Figure 2A,B, ESM S4). ODs formed a continuous plate of calcified tissue.

The last ODs were found in adult Javelin sand boa (*E. jaculus*). ODs were similar to other species of *Eryx*; with tiny bony elements covering the surface of the tail and the distal part of the body around 4 cm anterior to the cloaca. The pattern was regular resembling the distribution of the scales (Figure 2D, ESM S5). The highest OD density was on the lateral parts of the body. We did not find any support for the presence of ODs in the Black pipe snake (*C. melanotus*) and Rubber boa (*C. bottae*). Caudal vertebrae of the Black pipe snake were typical, diminishing toward the tip (Figure 2E, ESM S6). The neural arch is extremely depressed, haemapophyses are tiny and blunt in all caudal and cloacal vertebrae, and laterally directed pleurapophyses are very short and blunt throughout the caudal series. The posteriormost (approximately) three to four caudal vertebrae are fused. Vertebrae in the Rubber boa are highly modified and almost fill the volume of the entire tail (Figure 2F, ESM S7).

## 4 Discussion

We utilised the µCT technique to nondestructively investigate the internal anatomy of snake tails in species suspected of possessing dermal armour. ODs were found on the most distal part of the tail in two species of shield‐tailed snakes. Generally, shield‐tailed snakes (Uropeltidae), which are native to Peninsular India and Sri Lanka, are highly specialised snakes in various aspects of their life and some of those specialisations can relate to the presence of ODs. The tail is very short, and the tip is covered with a keratinous shield, giving rise to the moniker of shield‐tailed snakes. The shield has long been used as a taxonomic trait (Gower and Maduwage 2011; Ganesh et al. 2021; Gower et al. 2023, 2024), because its size, shape and arrangement of the ornamentation differ between genera (e.g., *Uropeltis*, *Rhinophis* and *Pseudotyphlops*). There are two main types of shields thoroughly described in Smith (1943), the disc‐like (flattening on the dorsal part with modifications of the scales), and the cylindrical‐like (terminating with a flattish or convex, round or oval rugose shield). Between spines and ridges covering the shield are settling grains of sand and clay, which form an initial layer of soil over the rough caudal surface (Gans 1976). It was observed that these dirt‐capped caudal shields share the colour and texture of the substratum and thus can work as a plug of the tunnels (Gans 1976). It was repeatedly hypothesised that such a plug can bring a significant selective advantage as it can successfully confuse and deter the predator that might follow a snake down a tunnel (McCann 1924; Gans 1976). Moreover, the shield can be underlain by a terminal bony process modified from the last caudal vertebra (Baumeister 1908; Huntley et al. 2021) in some species with cylindrical‐like shields (*Rhinophis planiceps, R. philippinus, R. trevelyanus*). Unfortunately, the anatomy and function of the modified last caudal vertebra are not well understood but it can certainly increase the strength of the tail tip. Our exploration of the tail tips of shield‐tailed snakes uncovers the presence of a terminal bony process in *R. homolepis* and one more bony layer situated between the terminal bony process and the shield. A similar bony layer was also found in *U. macrolepis*. It is placed again below the disk‐like shield. We hypothesise that this thin bony layer is a dermal armour and can together with the shield and modified caudal vertebrae work as a reinforcing element of the tail tip.

Several indications suggest that the caudal tip of shield‐tailed snakes is under considerable selective pressure. The short and rounded tail resembles the head (Gans 1986). Cephalic resemblance can be highly adaptive in combination with the antipredatory behaviour of shield‐tailed snakes. When attacked by predators, they display their tails while concealing their heads between their body coils (Gans 1986). Such behaviour targets the attacks toward the reinforced tail, increases the handling time for predators and consequently allows the snake to escape unharmed. Even though uropeltid snakes are nocturnal and fossorial, they are often ornamented (Cyriac and Kodandaramaiah 2019) on the ventral surface of the body with bright conspicuous colouration (yellow or red), which is uncovered when attacked. Although these snakes spend most of their life underground, they are occasionally foraging on the surface during the rainy season (Rajendran 1985). It was recently experimentally demonstrated that the conspicuous colouration of uropeltid snakes significantly reduces predation, possibly because these colours advertise unprofitability due to long handling times (Cyriac and Kodandaramaiah 2019). We hypothesise that the armoured tail in Uropeltidae serves a specific functional role, most likely providing mechanical protection against predators. Alternatively, it can occasionally also work as an anchoring mechanism to prevent the snake from being extracted from its tunnel or burrow. If the latter is correct, it would represent another example of phragmosis. On the other hand, in comparison with the function of ODs in *Eryx*, we can exclude the mechanical defence against combative prey as the diet of shield‐tailed snakes consists mostly of invertebrates, particularly earthworms (Smith 1943).

The anatomy of shield‐tailed snakes is highly modified due to the fossorial way of life. They are burrowing in moist forest regions with their wedge‐shaped keratinised head, which provides a great penetration tool for the extension of the tunnels (Gans et al. 1978). It was previously hypothesised that the shield on the tail tip could be involved as a lever in locomotion (Nicholls 1929). The anterior axial musculature consisting mostly of red muscles is prepared to exert great forces for a longer duration in comparison to white muscles in the posterior axis musculature. Biochemical analysis uncovered radical differences in the amounts of myoglobin, as well as qualitative and quantitative differences in enzymatic constituents (Proske and Ridge 1974) between anterior and posterior muscles. It was demonstrated that the anterior part of the body (25%–35% of the trunk) works as a propulsive system for burrowing and is well‐prepared for sustained activity (Gans et al. 1978). The bends of the neck (but not the tail) provide the base (so‐called point d'appui) for pushing the head through the soil. The movement along the tunnel is unidirectional (Gans et al. 1978). The posterior zones of the body are reserved for the visceral contents (Gans et al. 1978). The anterior part of the body can be compared to the locomotive, housing the primary propulsive mechanisms responsible for forward movement. This region pulls the relatively inert posterior part of the body and tail. Thus, based on the highly specialised type of locomotion, the presence of ODs is unlikely to be associated with movement within tunnels. However, observations of surface movements suggest that ODs may play a role when the snake begins to penetrate the substrate. At this initial stage, the neck is unable to contribute to the drilling process, but the armoured tail may function as a spade‐shaped anchor or base.

In this study, we also uncovered dermal armour in two individuals of Javelin sand boas (*E. jaculus*). We previously did not find ODs in this species (Frýdlová et al. 2023), nevertheless we had only one adult specimen. In this study, we had two larger individuals, which can explain our finding due to the tight correlation of OD development with the age and size of the animal. Unfortunately, it is hard to consider whether the development of ODs is also connected with the age of shield‐tailed snakes. First, our sample size is small, and second, information about the body growth and size of shield‐tailed snakes is limited. We found in the literature only very old and general statements about the total length and diameter of the body of currently explored shield‐tailed snakes (Smith 1943) but no data on maximal body length, sexual size dimorphism, body growth dynamics and so on. According to the literature, our specimens are rather subadults (65%–75% TOL_rel_).

We did not find ODs in Rubber boas (*C. bottae*), which were highly suspected of carrying them due to many similarities in morphology (highly modified caudal vertebrae described by Bogert 1968; tail resembling head, Stewart 1977) and behaviour (tail displays, Greene 1973; foraging tactics, Hoyer 1974) with sand boas. Their blunt tails with a high prevalence of injuries and scars (Hoyer and Stewart 2000a) are undoubtedly under strong pressure. While foraging in burrows upon broods of small mammals, which predominate in their diet (66% Rodríguez‐Robles et al. 1999), they use the lower part of the body and tail to ward off attacks from the defending parents (Hoyer and Stewart 2000a, 200b). Having dermal armour would be advantageous. Unfortunately, we only had one specimen of Rubber boa for exploration. Nevertheless, it was large enough (89% TOL_rel_) to carry at least ODs germs. Some snakes enhance their resistance to penetration by increasing the thickness of their integumentary layers. This adaptation has been observed in Calabar burrowing pythons (*Calabaria reinhardtii*), which possess a deep dermis that is more than seven times thicker than other snakes. Penetration experiments have demonstrated that thickened integument significantly improves their resistance to mechanical damage (Han and Young 2018). Even though they do not have ODs in their skin (Frýdlová et al. 2023), they have developed other passive defensive mechanisms to protect them against penetrative bites from maternal rodents. However, the mean thickness of the integumentary layers in Rubber boas is comparable to that of other snake species, so it does not give them any protective advantage (Han and Young 2018).

The reduction or complete absence of OD expression has traditionally been associated with subterranean lifestyle in squamate reptiles (Coe and Kunkel 1907; Camp 1923). Additionally, ODs have been suggested to limit flexibility during locomotion (Losos et al. 2002), offering an alternative or complementary explanation for their reduction or loss in elongated, limb‐reduced squamates. However, numerous exceptions challenge these general rules, such as preserved armour in caecilians (Zylberberg and Wake 1990), *Anguis* (Schmidt 1914), *Pseudopus* (Kéver et al. 2022) and *Heloderma* (Kirby et al. 2020). In addition, bearing armour is probably impossible for snakes because they need to have a flexible body due to the consumption of large prey. The position of ODs on the caudal region in snakes may represent a trade‐off between maintaining protective armour and preserving flexibility for locomotion and swallowing large prey. Alternatively, observations of captive‐bred subterranean snakes (e.g., *Calabaria*) suggest a different scenario. These snakes often refuse to accept prey out of enclosed spaces that simulate natural burrows (personal observation of D. F.), so it seems that they feel safer here. The cranial region can be actively defended through biting, while the mid‐body can rely on constriction or pressing opponents against the burrow walls to cause suffocation. Without passive mechanical protection, the tail becomes the most vulnerable body part. The presence of dermal armour in sand boas and shield‐tailed snakes likely results from a complex interplay and trade‐off between locomotion, foraging ecology, behaviour, predator interactions and threats from combative prey. We hypothesise that the primary function of the dermal armour in these groups is mechanical defence, though it may operate in different contexts.

## 5 Conclusions

In conclusion, we have uncovered three new species of snakes carrying dermal armour on their tail tips. In Javelin sand boa, the scenario of mechanical defence against aggressive prey is suspected. Conversely, a broader context for mechanical defence can be proposed in shield‐tailed snakes. The combination of highly specialised morphological adaptations and ecological characteristics in these secretive snakes suggests that the ODs can be involved in a dual function: antipredatory defence and locomotion.

## Author Contributions

Petra Frýdlová and Daniel Frynta—conceived and designed the research. Petra Frýdlová, Jan Dudák and Veronika Tymlová—curated the data and prepared the figures. Petra Frýdlová, Jan Žemlička, Jan Dudák and Veronika Tymlová—collected and analysed the data. Petra Frýdlová, Jan Dudák and Daniel Frynta—wrote the first draft of the manuscript. Petra Frýdlová, Jan Dudák, Veronika Tymlová, Jan Žemlička, Jiří Moravec and Daniel Frynta—writing and editing. All authors approved the final version of the manuscript.

## Conflicts of Interest

The authors declare no conflicts of interest.

### Peer Review

The peer review history for this article is available at https://www.webofscience.com/api/gateway/wos/peer-review/10.1002/jmor.70034.

## Supporting information

Supporting information.

Supporting information.

Supporting information.

Supporting information.

Supporting information.

Supporting information.

Supporting information.

## Data Availability

All data generated or analysed during this study are included in this published article's Electronic Supplementary Material (ESM [Link], [Link], [Link], [Link], [Link], [Link], [Link]) and on the figshare (http://figshare.com/s/281ff1668b16318ac01c).
