# Supplementary material for: Hidden Armour: The Passive Protective Function of Caudal Osteoderms in Snakes
Source: J Morphol. 2025 Feb 22;286(2):e70034. doi: 10.1002/jmor.70034 (PMC11846078; doi:10.1002/jmor.70034)
Supplement: Supplementary file 1 — Supporting information. [file JMOR-286-e70034-s001.docx]

**Electronic Supplementary Material 1**

**Hidden armour: The passive protective function of caudal osteoderms in snakes**

Petra Frýdlová, Jan Dudák, Veronika Tymlová, Jan Žemlička, Jiří Moravec, Daniel Frynta

**Supplementary Table 1.** CT scan parameters

| Specimen and sample ID | Beam settings kVp/uA | Beam filtering | Angle step (deg) | Number of projections | Voxel size (µm) |
| --- | --- | --- | --- | --- | --- |
| *Rhinophis homolepis* 73419 | 70/200 | 1 mm Al | 0.15 | 2400 | 5.0 |
| *Rhinophis homolepis* 73420 | 70/200 | 1 mm Al | 0.15 | 2400 | 5.6 |
| *Uropeltis macrolepis* 73421 | 70/200 | 1 mm Al | 0.15 | 2400 | 8.7 |
| *Charina bottae* 32794 | 70/200 | 1 mm Al | 0.15 | 2400 | 11.5 |
| *Cylindrophis melanotus* 401 | 70/100 | 1 mm Al | 0.15 | 2400 | 16.2 |
| *Eryx jaculus* 817 | 90/150 | 1 mm Al | 0.7 | 6497 | 12.5 |
| *Eryx jaculus* 818 | 90/150 | 1 mm Al | 0.6 | 4500 | 15.0 |

**Captions for supplementary videos**

**ESM 2.** Video file. Visualisation of osteoderms on the tail of subadult Travelyan´s earth snake (*Rhinophis homolepis*) ID 73419 by µCT. The last caudal vertebra (blue) is highly enlarged and modified. Dermal armour (yellow) is on the surface of the tail tip below the shield (not visible here). Dermal armour is a thin continuous layer of the calcified tissue copying the surface of the modified caudal vertebra. Author: Jan Dudák.

**ESM 3.** Video file. Visualisation of osteoderms on the tail of subadult Travelyan´s earth snake (*Rhinophis homolepis*) ID 73420 by µCT. The last caudal vertebra (blue) is highly enlarged and modified. Dermal armour (yellow) is on the surface of the tail tip below the shield (not visible here). Dermal armour is a thin continuous layer of the calcified tissue copying the surface of the modified caudal vertebra. Author: Jan Dudák.

**ESM 4**. Video file. Visualisation of osteoderms on the tail of subadult Bombay earth snake (*Uropeltis macrolepis*) by µCT. Caudal vertebrae of this species are rather typical as in other snake species (diminishing towards the tip). The last three vertebrae are fused. We did not observe any other specific caudal modifications. Dermal armour (yellow) was on the dorsal side of the tail directly below the tail shield (not visible here). Osteoderms were formed into a continuous plate of calcified tissue. Author: Jan Dudák.

**ESM 5**. Video file. The visualisation of osteoderms on the caudal part of the body of an adult Javelin sand boa (*Eryx jaculus*) by µCT. The small yellow structures are osteoderms, which are present predominantly on the lateral sides of the tail and partially on the caudal part of the body anterior to the cloaca. Osteoderms do not cover the body continuously; rather they are individually distributed across the surface of the skin. The distribution of osteoderms on the tail is regular resembling the distribution of scales. Author: Veronika Tymlová.

**ESM 6**. Video file. Visualisation of the caudal part of the body of a subadult of the Black pipe snake (*Cylindrophis melanotus*) by µCT. The caudal vertebrae of the Black pipe snake are not modified. They are diminishing towards the tail tip. No osteoderms were found. Author: Veronika Tymlová.

**ESM 7**. Video file. Visualisation of the tail of an adult Rubber boa (*Charina bottae*) by µCT. The caudal vertebrae are highly modified and almost fill the volume of the entire tail. No osteoderms were found. Author: Veronika Tymlová.
